# Breast and cervical cancer screening in a semi-urban African setting: evidence from Franceville, Gabon (2015–2021)

**DOI:** 10.3389/fonc.2026.1735802

**Published:** 2026-05-18

**Authors:** Pamela Moussavou-Boundzanga, Nathalie Ledaga Ambounda, Boris-Enock Zinsou, Berthold Bivigou-Mboumba, Moussa Togola, Augustin Mouinga-Ondeme, Ivan Mfouo-Tynga, Anne-Danielle Mavoungou-Niongo, Aime Roger Nzingou, Landry Eric Mombo, Cyrille Bisseye, Pascal Pineau

**Affiliations:** 1 Unité des Infections Rétrovirales et Pathologies Associées, Centre Interdisciplinaire de Recherches Médicales de Franceville, Franceville, Gabon; 2 Laboratoire de Biologie Moléculaire et Cellulaire, Université des Sciences et Techniques de Masuku, Franceville, Gabon; 3 Gynecology and Obstetric Service, University Hospital Centre of Libreville, Liberville, Gabon; 4 Université Paris Cité, IRD, Inserm, MERIT, Paris, France; 5 Unité Mixte de Recherche Centre International de Recherches Médicales de Franceville et Service de Sante, Militaire, Libreville, Gabon; 6 Service de gynécologie obstétrique, Centre Hospitalier Universitaire Amissa Bongo, Franceville, Gabon; 7 Institut Pasteur, Université Paris Cité, Unité “Virus & Stress Cellulaire”, Paris, France

**Keywords:** breast cancer, cancer screening, cervical cancer, clinical breast examination (CBE), Gabon, sub-Saharan Africa, visual inspection with acetic acid (VIA)

## Abstract

**Background:**

Breast and cervical cancers are among the most frequent malignancies in women worldwide and represent a growing public health concern in sub-Saharan Africa (SSA). In Gabon, national screening programs have been established, but their effectiveness remains poorly documented, particularly outside the capital city, Libreville. This study, therefore, aimed to describe the screening outcomes and associated factors for breast abnormalities and suspected cervical lesions at the Centre Hospitalier Universitaire Amissa Bongo (CHUAB) in Franceville, Gabon.

**Methods:**

We conducted a retrospective cross-sectional study that included all women who attended routine screening at CHUAB between 2015 and, 2016 and, 2018 and 2021. Data were retrieved from hospital registries and medical records. All women underwent both clinical breast examination (CBE) and visual inspection with acetic acid (VIA). Sociodemographic, reproductive, and clinical variables were analyzed to determine their associations with screening outcomes using univariable and exploratory multivariable logistic regression models.

**Results:**

A total of 1,328 women were included in the study, with a mean age of 35.4 ± 10.8 years. In univariable analysis, women aged 45–73 years had lower odds of cervical screening positivity compared with those aged 14–25 years (OR = 0.52, 95% CI: 0.29–0.90; p = 0.021). In exploratory multivariable analysis, non-Gabonese nationality and employment in the public sector were associated with cervical screening positivity. Regarding breast abnormalities, an age at first pregnancy between 30 and 39 years was associated with higher odds of screening positivity in univariable analysis (OR = 3.90, 95% CI: 1.07–11.4; p = 0.021); however, no factors remained statistically significant in the adjusted model.

**Conclusion:**

This study offers one of the first accounts of routine breast and cervical cancer screening outcomes in a semi-urban setting in south-eastern Gabon. VIA and CBE detected a measurable proportion of women with suspected cervical lesions and breast abnormalities, underscoring the value of these simple and accessible screening approaches in resource-limited settings. Strengthening follow-up systems, improving data management, and expanding screening coverage could further enhance cancer control among women in Gabon.

## 1 Introduction

Globally, breast and cervical cancer are among the most commonly diagnosed cancers in women and represent a major public health problem. The World Health Organization estimates that in 2022, approximately 666,000 women died from breast cancer and 350,000 from cervical cancer worldwide. A disproportionate number of these deaths occurred in Low and Middle Income Countries (LMICs) (around 71% for breast cancer, 91% for cervical cancer). A significant portion of these deaths could be prevented with increased public awareness and early-stage diagnosis (1).

The natural histories of breast and cervical cancers are interconnected, as they are both partly influenced by a woman’s sexual and reproductive choices, as well as exposures during early life (2). Consequently, a unified global approach to reducing these cancers is both feasible and necessary. In High-income countries (HICs), mortality from these cancers has been successfully reduced through organized screening strategies, including mammographic and cytology-based screening, often implemented through proactive public health policies.

By contrast, in LMICs, these trends are often reversed. Rapid changes in lifestyle and urban environments, driven by economic growth and the increasing participation of women in the workforce, have influenced breast cancer risk factors, including delayed childbearing, fewer children, excess weight, and physical inactivity. These evolving risk profiles are now aligning more closely with those of HICs (3, 4). As a result, in sub-Saharan African countries, both breast cancer incidence and mortality remain high, in part because of late-stage diagnosis and limited access to comprehensive treatment center services, leading to a poor prognosis for affected women (2).

Regarding cervical cancer, urbanisation and social changes are contributing to an earlier sexual debut and an increasing number of lifetime partners among women. In this context of lax screening policies, these factors contribute to increasing or at least maintaining the incidence of cervical cancer.

Inexpensive and feasible screening tests are available for these cancers. As recommended by the WHO, clinical breast examination (CBE) and visual inspection of the cervix after application of acetic acid (VIA) are suitable methods for early detection approaches in low-resource settings, particularly where mammography or cytology-based programmes remain difficult to implement at scale (5).

In Gabon, recent population-based cancer data from the Grand Libreville cancer registry showed that breast cancer is the leading cancer among women, followed by cervical cancer, together accounting for nearly half of all female cancers recorded in this urban setting (6). A national screening program for breast and cervical cancers, based on CBE and VIA, respectively, was established in Libreville, Gabon, in 2013. In 2015, authorities extended these screening services to Franceville, the capital of Haut-Ogooué province, located at the eastern border of the country, neighboring Congo. These units perform cervical and breast cancer screenings using visual inspection of the cervix and clinical breast examination. According to the Ministry of Health guidelines, women aged 20 years and older, as well as all sexually active women, are eligible for screening. However, to date, only partial data from Libreville, Gabon’s capital, have been published. Data from semi-urban and provincial settings remain scarce.

Thus, this study aims to provide data on women who attended the Franceville screening unit between 2015 and 2021 to assess the positivity rates of breast and cervical cancer screening and identify associated sociodemographic factors in the Haut-Ogooué province.

## 2 Materials and methods

### 2.1 Study area

The study was conducted at the Centre Hospitalier Universitaire Amissa Bongo (CHUAB) in Franceville, a semi-urban city and the provincial capital of Haut-Ogooué in southeastern Gabon. As the third most populous province, Haut-Ogooué is a region of significant mining activity, primarily for uranium and manganese. CHUAB is the main referral hospital in Haut-Ogooué and also serves as a regional referral centre for southeastern Gabon, receiving patients from Franceville and the surrounding areas, including neighboring localities.

The hospital hosts a dedicated screening unit where women may be referred or attend voluntarily for opportunistic screening. This unit serves women from Franceville and the surrounding areas within the province. In the absence of a fully organised population-based screening programme, screening activities at CHUAB represent an important entry point for the early detection of women’s cancers in this semi-urban, resource-constrained setting.

This setting is particularly relevant for the present study, as it provides access to routine screening data from a region of Gabon where published information on breast and cervical cancer screening remains limited.

### 2.2 Study design

This retrospective cross-sectional study was conducted at Centre Hospitalier Universitaire Amissa Bongo (CHUAB) in Franceville, which serves as the regional referral hospital and the main cancer screening center for the province of Haut-Ogooué. The study included the records of all women who attended the CHUAB screening unit for cervical and breast cancer screening during the study period (2015–2016 and 2018–2021). As part of the routine screening procedure, all women underwent both cervical cancer screening by visual inspection with acetic acid (VIA) and breast cancer screening by clinical breast examination (CBE).

Records were eligible if they contained screening information and could be matched to an individual patient file. Duplicate records and records with incomplete key information were excluded from the final analysis. The sample size corresponded to all eligible screening records available during the study period.

### 2.3 Study procedures and data collection

Data collection was conducted in two phases. First, information was extracted from the screening unit registry. Second, the corresponding individual medical records were retrieved and reviewed. Full names and registration numbers were used to match entries between the registry and patient files. To improve data reliability, extracted information was cross-checked between the registry and the individual medical records.

A standardized data extraction sheet was used to ensure consistency during data collection and to reduce transcription errors. Data were subsequently entered into a structured Microsoft Excel database, cleaned, and checked for consistency before statistical analysis. The following variables were collected: socio-demographics (screening site, year of survey, year of birth, age, nationality, origin, department, occupation, and marital status); family history variables (breast cancer, cervical cancer, diabetes, high blood pressure); obstetrical and reproductive history (pregnancy, parity, live child, abortion, menarche, age of first procedure) and screening test result.

Screening examinations were routinely performed by trained healthcare providers working in the breast and cervical cancer screening unit. For breast cancer, screening was based on clinical breast examination (CBE), including visual inspection and palpation of the breasts. A screening result was considered abnormal when a suspicious palpable mass, skin retraction, nipple discharge, or another clinically suspicious breast lesion was identified. For cervical cancer, screening was based on Visual Inspection after applying acetic acid (VIA). A cervical screening result was considered positive when acetowhite lesions suggestive of suspected cervical lesions were observed after acetic acid application.

Two screening outcomes were analysed separately in this study:

-cervical screening positivity, defined as a positive VIA result suggestive of suspected cervical lesions; and
-breast screening positivity, defined as an abnormal CBE suggestive of breast abnormalities.

After screening, the results were recorded on a standardized form and in the service registry. A copy of the form was provided to the patient.

The selection of patients’ records was conducted in two phases. First, the data were extracted from the unit registry. Second, we processed the individual medical records of each screened woman. The full names and registration numbers were used to identify and match records between the registry and patient files. To improve data reliability, the extracted information was cross-checked between the registry and the individual medical records. Duplicate entries and records with incomplete key information were excluded from the final analysis.

### 2.4 Data analysis

Data were analysed using RStudio. Categorical variables were summarized using counts and percentages, while continuous variables were described using means and standard deviations or medians and interquartile ranges, as appropriate. The proportion of missing data was assessed for all variables and reported in the descriptive analyses.

Associations between categorical variables and screening outcomes were assessed using Pearson’s chi-square test when expected cell counts were sufficient, and Fisher’s exact test when sparse data were present. Continuous variables were compared using either the Student’s t-test or the Mann–Whitney U test, depending on the distribution of the data. Correlations between continuous variables were assessed using Pearson’s or Spearman’s correlation coefficients, as appropriate.

For data processing, records with missing key screening outcome information were excluded from the analysis. For other variables, analyses were conducted using available data. For selected family history variables, missing values were recoded as “No” under the assumption that non-reporting reflected absence of known history; however, this approach may have introduced non-differential misclassification. Implausible values (below 10 years) for age at menarche and age at first pregnancy, representing less than 3% of observations, were considered invalid and treated as missing values. Variables with a high proportion of missing data (>50%) were handled with caution and were not prioritised in multivariable modelling.

Associations between explanatory variables and each screening outcome were first explored using univariable logistic regression models to estimate crude odds ratios (ORs) and their 95% confidence intervals (CIs).

Exploratory multivariable logistic regression models were then fitted separately for each screening outcome to assess independent associations. However, because of substantial missing data for several variables and sparse data in some categories, these models showed instability, including convergence issues and imprecise or non-interpretable estimates. Multivariable findings were therefore interpreted cautiously and considered exploratory rather than confirmatory. Geographic variables reflected screening locations rather than intrinsic individual characteristics and were therefore interpreted with caution as explanatory variables. Multiple imputation approaches were explored to address missing data, but did not substantially improve model stability. The primary analyses were therefore based on complete-case data. A p-value <0.05 was considered statistically significant.

### 2.5 Ethics statement

The study was carried out in accordance with the ethical principles outlined in the Declaration of Helsinki. Ethical approval was obtained from the institutional ethics committee of Centre Hospitalier Universitaire Amissa Bongo (CHUAB). As this was a retrospective study based on routinely collected screening records, no direct patient contact was involved. All data were anonymised before analysis, and confidentiality was strictly maintained throughout the study.

## 3 Results

### 3.1 Demographics of study participants

Initially, 1,479 medical files were recorded in the register. After removing duplicates and files without screening results, a total of 1,328 medical files were included in the study between 2015 and 2021. The main socio-demographic and reproductive features of the enrolled sample are displayed in **Tables 1** and **2**.

**Table 1:** Sociodemographic and reproductive factors associated with cervical cancer screening positivity.

Characteristic		Cervical cancer		p-value
		Negative (N = 368)	Positive (N = 38)	
**Age**	[14-25]	12% (44)	7.9% (3)	0.696
	[25-35]	35% (130)	42% (16)	
	[35-45]	25% (93)	29% (11)	
	[45-73]	27% (101)	21% (8)	
**Nationality**	Gabonese	95% (349)	89% (34)	0.255
	Non-gabonese	5.2% (19)	11% (4)	
**Marital status**	Unmarried	61% (224)	58% (22)	0.855
	Married	39% (144)	42% (16)	
**City of screening**	Franceville	68% (252)	74% (28)	0.790
	Leconi	1.4% (5)	0% (0)	
	Moanda	7.6% (28)	2.6% (1)	
	Others	23% (83)	24% (9)	
**Department**	Lemboumbi-Leyou	8.7% (32)	5.3% (2)	0.629
	Mpassa	82% (301)	89% (34)	
	Others	9.5% (35)	5.3% (2)	
**Province of screening**	Haut-Ogooué	97% (357)	95% (36)	0.348
	Others	3.0% (11)	5.3% (2)	
**Age at menarche**	[7-12]	3.3% (12)	0% (0)	0.682
	[12-16]	74% (272)	74% (28)	
	[20- +]	23% (84)	26% (10)	
**Pregnancy**	Yes	100% (368)	100% (38)	p=<0.001
**Age at first pregnancy**	10-19	63% (231)	55% (21)	0.456
	20-29	35% (127)	45% (17)	
	30-39	2.7% (10)	0% (0)	

P-values were calculated using Fisher’s exact test when expected frequencies were <5; otherwise, the Chi-square test was used.

**Table 2:** Sociodemographic and reproductive factors associated with breast cancer screening positivity.

Characteristic			Breast cancer	p-value
		Negative (N = 439)	Positive (N = 21)	
Age	[14-25]	12% (51)	9.5% (2)	0.477
	[25-35]	33% (145)	43% (9)	
	[35-45]	28% (125)	14% (3)	
	[45-73]	27% (118)	33% (7)	
Nationality	Gabonese	93% (409)	95% (20)	p=1.000
	Non-gabonese	6.8% (30)	4.8% (1)	
Marital status	Unmarried	60% (262)	57% (12)	p=0.997
	Married	40% (177)	43% (9)	
City of screening	Franceville	66% (289)	86% (18)	0.280
	Leconi	1.6% (7)	0% (0)	
	Moanda	11% (49)	0% (0)	
	Others	21% (94)	14% (3)	
Department	Lemboumbi-Leyou	13% (55)	0% (0)	0.122
	Mpassa	79% (345)	86% (18)	
	Others	8.9% (39)	14% (3)	
Province of screening	Haut-Ogooué	97% (425)	95% (20)	0.509
	Others	3.2% (14)	4.8% (1)	
Age at menarche	[7-12]	3.4% (15)	0% (0)	0.572
	[12-16]	73% (321)	86% (18)	
	[20- +]	23% (103)	14% (3)	
Pregnancy	Yes	100% (439)	100% (21)	p=<0.001
Age at first pregnancy	10-19	62% (272)	62% (13)	0.150
	20-29	36% (157)	29% (6)	
	30-39	2.3% (10)	9.5% (2)	

Overall, most women were aged 25–35 years, Gabonese, and screened in Franceville, mainly in the Mpassa department. Most participants reported a first pregnancy between 10 and 19 years and menarche between 12 and 16 years. The distribution of sociodemographic and reproductive characteristics was broadly similar between women with negative and positive screening results for both cancers. Suspected cervical lesions and breast abnormalities were detected among 9.36% and 4.56% women, respectively.

### 3.2 Sociodemographic and reproductive factors associated with suspected cervical lesions

#### 3.2.1 Univariable analysis

To further explore factors associated with cervical screening positivity, univariable logistic regression analyses were performed for selected sociodemographic and reproductive characteristics (**Table 3**). In univariable analysis, age was the only variable significantly associated with suspected cervical lesions.

**Table 3:** Univariable logistic regression analysis of factors associated with cervical cancer screening positivity.

Characteristic		N	OR	95% CI	p-value
Age	[14-25]	1,017	Reference	—	—
	[25-35]		0.88	0.56, 1.39	0.6
	[35-45]		0.90	0.56, 1.47	0.7
	[45-73]		0.52	0.29, 0.90	**0.021**
Nationality	Gabonese	869	Reference	—	—
	Non-gabonese		1.17	0.47, 2.50	0.7
Marital status	Unmarried	1,002	Reference	—	—
	Married/engaged/widowed		0.85	0.60, 1.19	0.4
City of screening	Others	1,048	—	—	
	Franceville		0.83	0.57, 1.23	0.3
	Leconi		0.34	0.02, 1.82	0.3
	Moanda		0.81	0.39, 1.60	0.6
Occupation	nactive/unemployed	909	—	—	
	Skilled professions		0.98	0.56, 1.65	>0.9
	Informal/self-employed sector		0.64	0.19, 1.67	0.4
	Public sector		1.20	0.77, 1.84	0.4
Age at menarche	[7-12]	825	—	—	
	[12-16]		1.44	0.60, 4.29	0.5
	[20- +]		1.74	0.68, 5.37	0.3
Pregnancy	No	827	—	—	
	Yes		2.28	1.10, 5.56	**0.043**
Age at first pregnancy (years)	10–19	638		—	—
	20–29		1.16	0.57, 2.34	0.7
	30–39		0.00		>0.9
Family history of cancer	No	1,048	Reference	—	
	Yes		0.68	0.16, 2.01	0.5
Family history of cervical cancer	No	1,048	—	—	
	Yes		1.11	0.25, 3.50	0.9

CI, confidence interval; OR, odds ratio. Bold values indicate statistically significant associations (p < 0.05).

Compared with women aged 14–25 years, those aged 45–73 years had significantly lower odds of a positive screening result (OR = 0.52, 95% CI: 0.29–0.90; p = 0.021). No statistically significant associations were found for age at first pregnancy, nationality, place of screening, professional status, marital status, age at menarche, or family history of cancer (all p > 0.05).

Although having had at least one pregnancy was associated with higher odds of a positive screening result (OR = 2.28, 95% CI: 1.10–5.56; p = 0.043), this variable was retained in the results but not emphasized in the interpretation, as all women with a positive screening result had experienced at least one pregnancy.

#### 3.2.2 Multivariable analysis

Variables showing evidence of association in univariable analysis, as well as those considered epidemiologically relevant, were subsequently entered into the multivariable logistic regression model (**Table 4**). In the adjusted model, non-Gabonese nationality and professional status remained significantly associated with cervical cancer screening positivity.

**Table 4:** Univariable logistic regression analysis of factors associated with breast cancer screening positivity.

Characteristic		N	OR	95% CI	p-value
Age	[14-25]	1,177	—	—	
	[25-35]		1.14	0.56, 2.46	0.7
	[35-45]		0.87	0.39, 1.98	0.7
	[45-73]		0.86	0.37, 2.02	0.7
Nationality	Gabonese	967	—	—	
	Non-gabonese		1.25	0.37, 3.25	0.7
Marital status	Unmarried	1,162	—	—	
	Married/engaged/widowed		0.72	0.41, 1.23	0.2
City of screening	Others	1,209	—	—	
	Franceville		1.37	0.71, 2.91	0.4
	Leconi		2.62	0.38, 11.0	0.2
	Moanda		0.61	0.14, 2.05	0.5
Occupation	nactive/unemployed	1,056	—	—	
	Skilled professions		0.73	0.25, 1.75	0.5
	Informal/self-employed sector		0.42	0.02, 2.00	0.4
	Public sector		1.51	0.80, 2.74	0.2
Age at menarche	[7-12]	967	—	—	
	[12-16]		0.55	0.22, 1.64	0.2
	[20- +]		0.39	0.13, 1.34	0.11
Pregnancy	No	970	—	—	
	Yes		0.73	0.33, 1.96	0.5
Age at first pregnancy (years)	10–19	757	—	—	
	20–29		1.03	0.52, 1.97	>0.9
	30–39		3.90	1.07, 11.4	**0.021**
Family history of cancer	No	1,209	—	—	
	Yes		1.05	0.17, 3.57	>0.9
Family history of breast cancer	No	1,209	—	—	
	Yes		0.68	0.04, 3.30	0.7

CI, confidence interval; OR, odds ratio. Bold values indicate statistically significant associations (p < 0.05).

Women of non-Gabonese nationality had higher odds of a positive screening result compared with Gabonese women (OR = 3.63, 95% CI: 0.90–12.4; p = 0.049). Likewise, women employed in the public sector had higher odds of screening positivity than inactive or unemployed women (OR = 2.44, 95% CI: 1.08–5.52; p = 0.031). No statistically significant associations were observed for the remaining variables.

### 3.3 Sociodemographic and reproductive factors associated with breast abnormalities

#### 3.3.1 Univariable analysis

Univariable logistic regression analyses were performed to assess factors associated with breast screening positivity (**Table 5**). Age at first pregnancy was the only variable significantly associated with screening positivity. Women who had their first pregnancy between 30 and 39 years had higher odds of a positive screening result compared with those aged 10–19 years (OR = 3.90, 95% CI: 1.07–11.4; p = 0.021). No statistically significant associations were found for the other sociodemographic and reproductive variables included in the analysis.

**Table 5:** Multivariable logistic regression analysis of factors associated with cervical cancer screening positivity.

Characteristic		OR	95% CI	p-value
(Intercept)	0.05	0.01, 0.17	**<0.001**	**(Intercept)**
Age	[14-25]	—	—	
	[25-35]	1.39	0.41, 6.44	0.6
	[35-45]	1.51	0.40, 7.33	0.6
	[45-73]	0.90	0.23, 4.47	0.9
Nationality	Gabonese	—	—	
	Non-gabonese	3.63	0.90, 12.4	**0.049**
Marital status	Unmarried	—	—	
	Married/engaged/widowed	0.93	0.44, 1.93	0.9
City of screening	Others	—	—	
	Franceville	1.24	0.57, 2.93	0.6
	Leconi	0.00		>0.9
	Moanda	0.41	0.02, 2.48	0.4
Occupation	nactive/unemployed	—	—	
	Skilled professions	1.61	0.59, 4.08	0.3
	Informal/self-employed sector	0.00		>0.9
	Public sector	2.44	1.08, 5.52	**0.031**
Age at first pregnancy (years)	10–19	—	—	
	20–29	1.16	0.57, 2.34	0.7
	30–39	0.00		>0.9
Family history of cancer	No	—	—	
	Yes	2.94	0.39, 14.5	0.2

CI, confidence interval; OR, odds ratio. Bold values indicate statistically significant associations (p < 0.05).

#### 3.3.2 Multivariable analysis

In the multivariable analysis (**Table 6**), no factor remained significantly associated with breast screening positivity. Women who had their first pregnancy between 30 and 39 years had higher odds of a positive screening result compared with those who had their first pregnancy between 10 and 19 years, although this association did not reach statistical significance (adjusted OR = 4.29, 95% CI: [0.56-22.0], p = 0.10). No statistically significant associations were observed for the other variables included in the model.

**Table 6:** Multivariable logistic regression analysis of factors associated with breast cancer screening positivity.

Characteristic		OR	95% CI	p-value
	**(Intercept)**	0.05	0.01, 0.17	**<0.001**
Age	[14-25]	—	—	
	] 25-35]	1.27	0.37, 5.90	0.7
	] 35-45]	0.33	0.05, 1.92	0.2
	] 45-73]	1.19	0.31, 5.83	0.8
Nationality	Gabonese	—	—	
	Non-gabonese	0.66	0.09, 2.88	0.6
Marital status	Unmarried	—	—	
	Married/engaged/widowed	1.16	0.47, 2.75	0.7
City of screening	Others	—	—	
	Franceville	1.44	0.56, 4.45	0.5
	Leconi	0.00		>0.9
	Moanda	0.00		>0.9
Age at first pregnancy (years)	10–19	—	—	
	20–29	0.73	0.27, 1.77	0.5
	30–39	4.29	0.56, 22.0	0.10
Family history of cancer	No	—	—	
	Yes	1.87	0.27, 7.68	0.4

CI, confidence interval; OR, odds ratio. Bold values indicate statistically significant associations (p < 0.05).

## 4 Discussion

Gabon is a country located within the historically described “infertility belt” of Africa. For decades perhaps even centuries the proportion of women without children in the country has remained notably high (7) (8). Reproductive health among young women, particularly conditions affecting reproductive health, including female cancers, is a matter of significant concern, as these conditions are often discussed among the factors that may contribute to reduced fertility patterns. This cross-sectional study reports on female cancer screening experiences in a semi-urban context in southeastern Gabon.

This is the first study to assess screening positivity rates and related sociodemographic factors for breast and cervical cancer screening in a semi-urban Gabonese province, Haut-Ogooué. These findings reflect data from the routine screening activities conducted at CHUAB programme within this province. They complement recent population-based cancer data from Gabon, which identified breast and cervical cancers as the two leading cancers among women in the Grand Libreville area, together accounting for approximately half of female cancers (6). The diverse profiles (nationality, department of origin, age, and professional status) of the women who participated demonstrate that the screening programme successfully reached women from various social backgrounds. These initiatives can improve early cancer detection. When combined with subsequent diagnostic confirmation and effective treatment, they have the potential to reduce the burden of female cancers in Gabon and, by extension, to contribute to improving women’s health outcomes in this broader regional context tire.

To strengthen early detection of women’s cancers in this setting, a simple screening programme based on visual inspection with acetic acid (VIA) for cervical cancer and clinical breast examination (CBE) for breast cancer was implemented. These approaches are particularly relevant in low-resource settings because they require limited equipment and can be delivered with relatively modest technical capacity, making them feasible options for expanding access to early detection services forward programme (5).

In our study, the overall screening positivity rate was 9.36% for suspected cervical lesions and 4.56% for breast abnormalities. The VIA positivity observed in our setting is comparable to the pooled African estimate of 11.93% reported in a recent meta-analysis, suggesting that the burden of abnormal cervical screening findings in our population is consistent with that reported elsewhere on the continent (9). Similarly, the proportion of women with breast abnormalities in our study was of the same order of magnitude as the 5.6% reported in a recent Tanzanian study based on abnormal clinical breast examination findings (10) (11) (12). These findings should also be interpreted in light of recent population-based cancer data from Gabon. In the Grand Libreville cancer registry, breast cancer ranked first and cervical cancer second among female cancers, together accounting for approximately half of all cancers diagnosed in women. Although our study assessed screening positivity rather than confirmed cancer diagnoses, this national context reinforces the public health relevance of maintaining early detection strategies for both conditions in Gabon (6).

These comparisons should nevertheless be interpreted with caution, as the observed outcomes reflect screening-detected abnormalities rather than confirmed cancer diagnoses. VIA and CBE are intended to identify women requiring further diagnostic evaluation, and their positivity rates may vary according to the underlying risk profile of the screened population, the organisation of screening activities, and local clinical practices.

Another notable finding was the relatively young age of women with positive screening results, particularly for suspected cervical lesions. This pattern is consistent with reports from several sub-Saharan African settings, where abnormal cervical screening findings are frequently observed among women in their thirties. In our study, women aged 25–35 years represented the largest proportion of screened women and a substantial proportion of those with positive results. Breast abnormalities were also observed in relatively young women. For breast screening, although direct comparisons remain limited because many published African data are based on confirmed breast cancer cases rather than screening abnormalities, our findings support the relevance of early detection initiatives targeting relatively young women in this context (13) (14).

Only a limited number of variables were associated with screening positivity in this study. In univariable analysis, older age (45–73 years) was associated with lower odds of cervical screening positivity, while age at first pregnancy between 30 and 39 years was associated with higher odds of breast screening positivity. In exploratory multivariable analysis, non-Gabonese nationality and employment in the public sector remained associated with cervical screening positivity, whereas no factor remained significantly associated with breast abnormalities.

### 4.1 Limitations

This study has several limitations. First, the absence of a digital database limited our ability to assess follow-up after screening and document subsequent patient the management program. As a result, we could not determine whether women with positive screening results completed confirmatory investigations or received appropriate care. Story program. Second, missing data for certain years (including 2017), and on the use of paper-based records, may have introduced incomplete reporting and potential information bias. Finally, as this was a single-center study, the findings may not fully reflect the situation in other provinces of Gabon, although CHUAB serves a broader catchment area in southeastern Gabon.

### 4.2 Perspectives and recommendations

To improve participation in screening, community awareness efforts and access to affordable screening services should be strengthened program. In addition, digital follow-up tools and mobile health innovations could improve documentation, continuity of care, and long-term monitoring of screened women. Strengthening these components may help improve the effectiveness and sustainability of breast and cervical cancer screening programmes in Gabon.

## 5 Conclusion

This study provides one of the first descriptions of routine breast and cervical cancer screening outcomes in a semi-urban setting of southeastern Gabon. VIA and CBE identified a measurable proportion of women with suspected cervical lesions and breast abnormalities, highlighting the value of these simple screening approaches in low-resource settings. Strengthening screening coverage, ensuring follow-up after abnormal findings, and improving digital health information systems will be essential to enhance early detection and women’s cancer control in Gabon.

## Data Availability

The original contributions presented in the study are included in the article/supplementary material. Further inquiries can be directed to the corresponding author.
